# Applying shot boundary detection for automated crystal growth analysis during in situ transmission electron microscope experiments

**DOI:** 10.1186/s40679-016-0034-x

**Published:** 2017-01-03

**Authors:** W. A. Moeglein, R. Griswold, B. L. Mehdi, N. D. Browning, J. Teuton

**Affiliations:** 1 National Security Directorate, Pacific Northwest National Laboratory, 902 Battelle Boulevard, Richland, WA 99352 USA; 2 Joint Center for Energy Storage Research (JCESR), Pacific Northwest National Laboratory, 902 Battelle Boulevard, Richland, WA 99352 USA; 3 Physical and Computational Sciences Directorate, Pacific Northwest National Laboratory, 902 Battelle Boulevard, Richland, WA 99352 USA; 4 Earth and Biological Sciences Directorate, Pacific Northwest National Laboratory, 902 Battelle Boulevard, Richland, WA 99352 USA; 5 Materials Science and Engineering, University of Washington, Seattle, WA 98195 USA

**Keywords:** Shot boundary detection, Electrochemistry, In situ transmission electron microscopy, Nucleation and growth, Big data analytics

## Abstract

In situ scanning transmission electron microscopy is being developed for numerous applications in the study of nucleation and growth under electrochemical driving forces. For this type of experiment, one of the key parameters is to identify when nucleation initiates. Typically, the process of identifying the moment that crystals begin to form is a manual process requiring the user to perform an observation and respond accordingly (adjust focus, magnification, translate the stage, etc.). However, as the speed of the cameras being used to perform these observations increases, the ability of a user to “catch” the important initial stage of nucleation decreases (there is more information that is available in the first few milliseconds of the process). Here, we show that video shot boundary detection can automatically detect frames where a change in the image occurs. We show that this method can be applied to quickly and accurately identify points of change during crystal growth. This technique allows for automated segmentation of a digital stream for further analysis and the assignment of arbitrary time stamps for the initiation of processes that are independent of the user’s ability to observe and react.

**Electronic supplementary material:**

The online version of this article (doi:10.1186/s40679-016-0034-x) contains supplementary material, which is available to authorized users.

## Background

Atomic-scale images of interfaces/defects obtained from scanning transmission electron microscopes (STEM) have long been used to provide insights into the structure–property relationships of materials—for example, observations of atomic-scale intermixing at interfaces in semiconducting/oxide heterostructures have helped understand the unique electronic and magnetic properties of these systems [1, 2]. The development and application of the STEM techniques used in these and other studies (for example, [3–9]) start from the premise that the atoms in the structure do not move. However, the systems that are being developed for many novel energy technologies are far removed from this paradigm—*their intrinsic functionality is wholly dependent on the motion of atoms*. For example, in Li-ion batteries, the charge/discharge cycle involves the mobility of ions across the electrolyte–electrode interface [10]. To identify the key aspects of the complex processes and transients occurring in energy technologies, we must therefore develop in situ or *operando* methods that allow us to observe directly the functions of the system taking place during operation of the device.

**Fig. 1:**
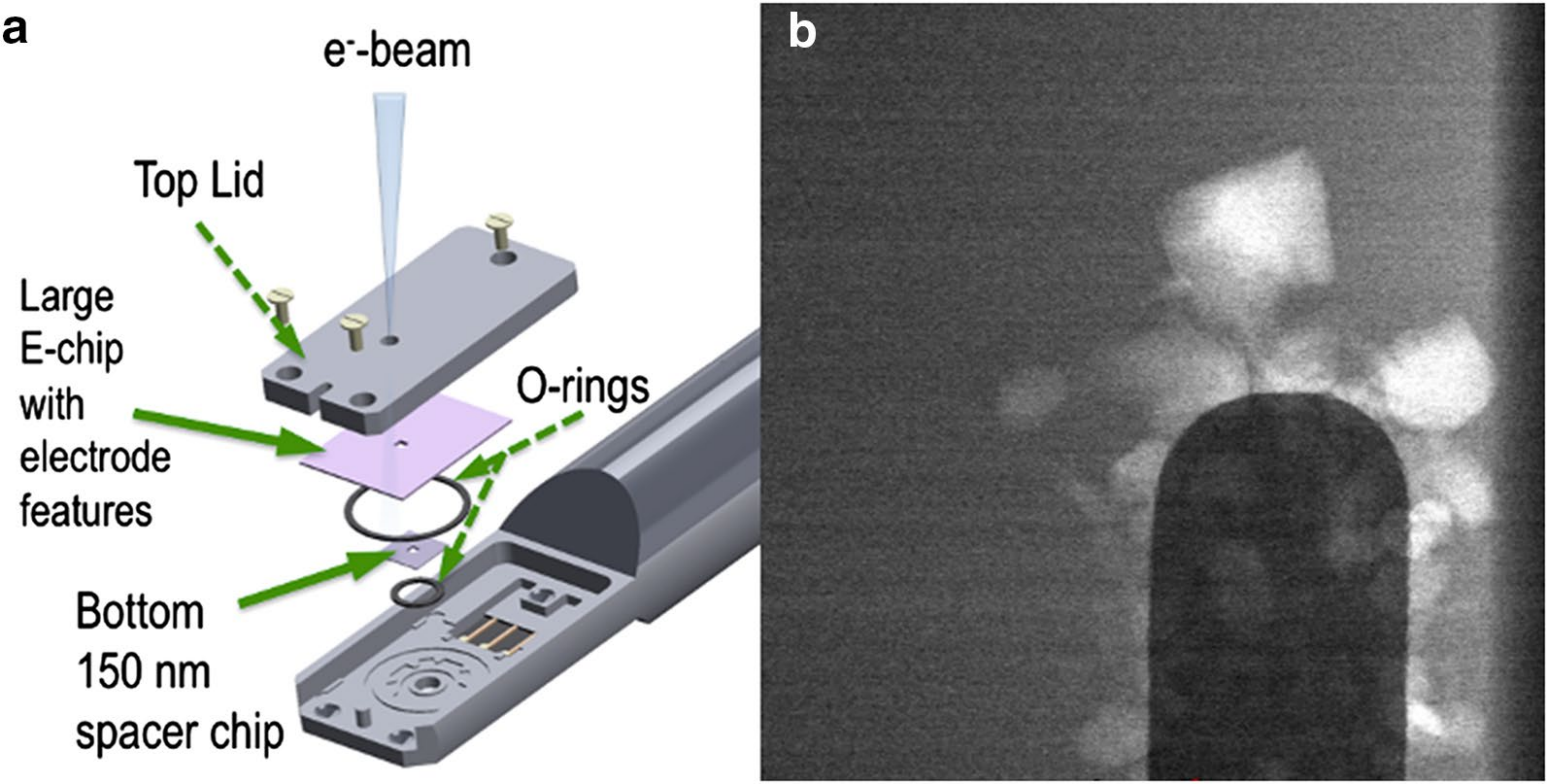
**a** Schematic of the operando nanobattery and **b** high-angle annular dark-field, HAADF, image frame from the movie of the electrodeposited Li on a Pt electrode in 1M LiPF_6_ in PC electrolyte (in a background) [7, 8]

For *operando* studies of electrochemical reactions, inside the in situ stages developed for STEM shown in Fig. 1a allow electrodes and a high-vapor pressure liquid electrolyte to be incorporated into the microscope [11–15], essentially forming a nanobattery. In these experiments, the images are recorded on either charge-coupled devices (CCDs) or direct detection complementary metal oxide semiconductor (CMOS) devices that have arrangements of pixels from 1 k × 1 k up to 4 k × 4 k. Understanding the electrochemical process involves scientists being able to directly image the initial stages of electrodeposition/nucleation at the electrode surfaces (the formation of Li dendrites). In current detectors, the frame rates are typically video rate (33 frames per second) with the more advanced cameras operating at 1000 frames per second. Future developments in both the microscopes and the detectors are expected to push this frame rate up by several orders of magnitude. Hence, the data challenge for analysis from a region of interest is already significant and promises to push the limits of what can be done very soon.

Current image capture and analysis is performed manually—the user starts the camera and looks for any change to occur in the images as they are recorded. This is a time-consuming process that requires frames to be individually analyzed to identify regions of interest. However, this type of problem—the identification of where and when in a series of frames there is a change—lends itself to automation. Recent trends in digital and streaming media have rapidly introduced a number of techniques that can be used to automate the analysis of videos [16]. These techniques have become increasingly important to streaming content providers looking to improve video search, indexing, and retrieval. In order to perform automated analysis of video, it is typically segmented into a hierarchy of shots. Shots refer to a group of frames that make up a single camera action. This process, referred to as shot boundary detection (SBD), allows for further analysis of digital media by regions of similar content. Computational efficiency is crucial to video segmentation in order to provide timely feedback. Previous work has been performed to evaluate the performance of segmentation techniques based on the video domain, type of transition, and type of detection feature [17–19]. This provides a baseline for choosing and evaluating suitable techniques for the type of data typically produced by STEM.

Video is typically stored and transmitted in a compressed format, such as one of the moving picture experts group (MPEG) standards. While these compressed formats are convenient for storage and streaming, they are computationally expensive to decompress for the purposes of analysis [20]. In the case of STEM where image data are captured at a rate of hundreds or thousands of frames per second, the expense of decoding the video grows very quickly. In this case, performing analysis of the compressed stream directly becomes an attractive option to increase efficiency. In this paper, we demonstrate the use of performing analysis on the compressed data stream. The example we use is the identification of the electrodeposition of Li during charge/discharge of a Li battery. The example identifies the onset of the deposition/first nucleation stages of Li metal that can be correlated with a specific voltage value controlling these changes. The potential to extend this form of compressed analysis to also identify where in the frame the process take place first (adding a spatial coordinate to the temporal one) will also be discussed.

## Methods

### Experimental

The in situ electrochemical STEM experiments were performed on a FEI 80–300 kV Cs-corrected Titan microscope equipped with Schottky field-emission electron source, a monochromator, and a CEOS hexapole spherical probe aberration corrector. For these experiments, the microscope was operated at 300 keV in both bight-field (BF) and high-angle annular dark-field **(**HAADF) modes (Fig. 1b; Additional file 1). All images were obtained after calibration of the dose, and the dose was kept below ≤0.3 electrons/Å^2^/s to avoid beam damage effects. All the electrochemical measurements were performed with a commercially available Poseiden 500 (Protochips Inc., Raleigh, NC, USA) microfluidic in situ electrochemical stage, which allows for simultaneous observation of dynamic electrochemical measurements in the liquid environment. Figure 1a illustrates an in situ liquid electrochemical scanning transmission (STEM) cell (*ec*-STEM) used for Li dendrite deposition/stripping in 1M LiPF_6_ in PC electrolyte with trace amount of water as shown in Fig. 1b. The in situ liquid *ec*-STEM cell is made from two silicon microchips containing 50-nm-thick silicon nitride membranes transparent to the electron beam and three Pt microelectrodes, aligned parallel to each other. The top electrochemical microchip has 500 nm SU-8 spacer and the bottom microchip has 150 nm gold spacer giving a nominal spacing of 650 nm. The electron beam passes through the electrolyte and two Si_x_N_y_ membranes allowing for recording the process of the Li dendrite growth and dissolution in *real*-*time* at high spatial and temporal resolution during cyclic voltammetry or galvanostatic charge/discharge process in both TEM and STEM modes at 2–3 µL/min flow rate. All the cycling voltammetry experiments were conducted with a Gamry Reference 600 potentiostat, and synchronized with simultaneous recording of the video sequence of Li dendrite deposition/dissolution process at the Pt electrode from LiPF_6_ in PC electrolyte in the in situ *ec*-STEM cell.

### Video streaming

Many techniques exist that aim to directly handle compressed video streams for quick and efficient processing. These techniques rely on the reduced signal and coefficients produced as part of the compression process [21]. The coefficients generated directly relate to the original uncompressed signal and can be used to detect transitions in a video. While there are numerous ways for video frames (scenes) to transition, they can typically be categorized as either a cut or gradual transition [22]. A cut occurs when a scene is ended in one frame and a new scene begins in the next frame. Gradual transitions are a change between two scenes where the content of one shot is slowly replaced with that of the next over several frames. Both of these types of shot boundaries can come in many different forms. In the case of crystal growth detection, we expect that after the initial nucleation event (a cut scene), a gradual transition will then take place as the material grows (this makes the gradual transition the most common technique and hence the primary focus of this work). An added complication for this type of experiment is that the object of focus (here size and shape of Li grains) tends to change over the course of several frames as the experiment is performed. With these types of gradual transitions, it is important to consider differences over a window of time. The window size varies depending on the speed and type of the transition. A general window size can be chosen to fit the transition type as well as the type of data observed.

#### MPEG standard

The MPEG standard provides a set of guidelines for video compression and transmission of video at a variable bitrate. The standard makes use of two techniques to achieve compression: a block-based motion compensation and the discrete cosine transform (DCT) [23]. These techniques take advantage of the spatial and temporal redundancy within a sequence of frames to reduce the amount of data necessary to reconstruct the video. The foundational component of a video is a frame. A frame is an image of a width and height that represents one step in a video. These frames often contain regions of similar visual content within themselves. Storing the values for each individual pixel in an image is costly and unnecessary. To eliminate these redundant data, the image is divided into small blocks called macroblocks (MB), to which the DCT is applied. The transformation produces a matrix of coefficients that represent each block of data. In order to further minimize the amount of data stored, an additional technique called quantization is applied [24]. Quantization reduces the transformation coefficient data to the smallest possible amount necessary to reconstruct each block. This additional step is designed to limit the frequencies stored for the image while reducing many of the frequency components to zero for optimal compression.

A video is composed of a series of frames, which when played back at a certain frame rate provide a visually fluid motion. Frames in a video typically have common data between one or more frames. To eliminate the need to store this content for individual frames, special frames called prediction frames are used [25]. These prediction frames (P-frames) reference other frames or MB within a frame which can be found before or after the predicted frame. Frames that do not reference other frames are referred to as intra-coded frames (I-frames). Frame references are calculated during a phase of the encoding process called motion estimation. The result of the motion estimation step is a model called the motion vector that describes the offset of coordinates shared between prediction and reference frames [26].

#### Types of video transitions

**Fig. 2:**
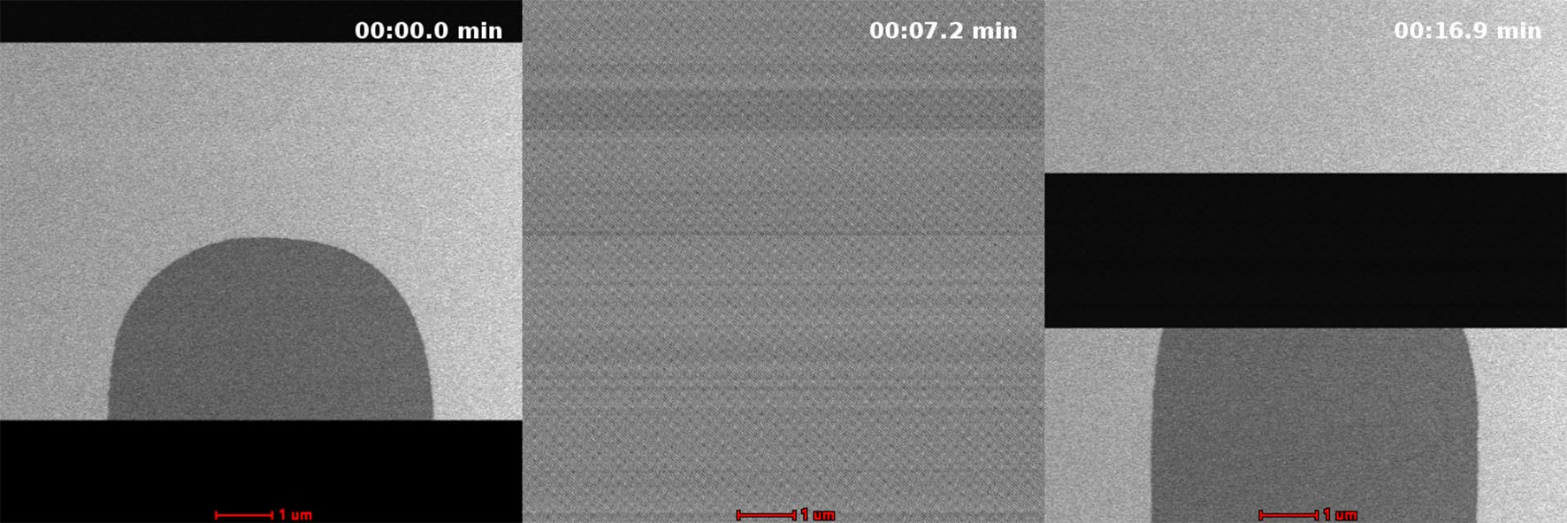
An abrupt shot transition is seen when adjusting focus [8]

**Fig. 3:**
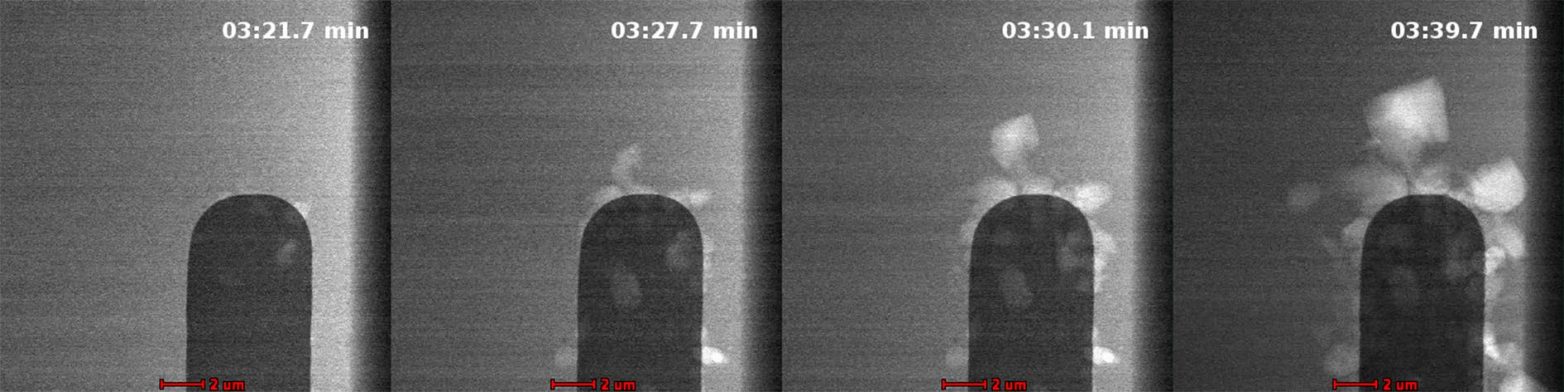
A gradual shot transition is seen as growth occurs [8]

Shot boundary detection is used to segment videos into different shots. Shots within a video are sequences of frames that make up a single camera action. Shot transitions are generally categorized as a hard cut or gradual transition. Hard cuts occur when two consecutive frames form the boundary between shots. The frames in Fig. 2 show an example of a hard cut; these neighboring frames have no similar content shared between them. These are easily detectable as there is little to no similarity between adjacent frames [27]. Gradual transitions take place over multiple frames and can have many different effects. The number of transition types with varying duration can make it difficult to detect [28]. Traditional videos contain a number of types such as pans, zooms, fades, and dissolves that have differing transitional characteristics. For the purpose of identifying the grain growth, the focus will be on dissolves. Dissolves occur when the contents of one shot transition to the next over some period where the shots overlap. The sequence of frames in Fig. 3 shows the transition typically found with grain growth. The transition occurs over multiple frames as the grain begins to form.

#### Encoding information

The videos used here have been encoded using the MPEG-2 standard. The MPEG-encoding process generates a number of statistics for each frame of a video. The encoding information can be accessed by partially decoding the compressed video. Partially decoding the video eliminates the need to calculate the original frame pixel values. The inverse transform performed for full decoding has been found to consume as much 40% of total decoding time [29]. Therefore, partial decoding results in a significant time savings over other methods. For the purposes of this paper, the FFmpeg library [30] is used to process and decode video streams. Shot boundary detection in the compressed domain makes use of features derived from the reduced signal to find change. Two types of features that can be used in change detection are frame and motion information [31]. The frame information refers to the type of frame encoding, such as I-frame or P-frame. This is important for decision making due to the different characteristics of each type of frame. Motion information includes the motion vector as well as MB motion features, such as the sum of variance (SoV). The SoV of each MB is used by the encoder to measure the amount of motion within the MB. This MB motion information is used by the encoder not only determines how the MB will be encoded, but also serves as an indicator of the amount of change occurring within each block.

#### Frame motion

With the encoded video, the frame and motion information can be extracted. Separate analysis of frames based on the frame type is carried out to take advantage of characteristics specific to each type. As previously discussed, predicted frames contain motion information which varies in size depending on the degree of change. Compared to P-frames, intra-coded frames (I-frames) have minimal motion information due to their limited relation to other frames. Motion information can be used to characterize the amount of change occurring within a frame. Scenes will have different motion levels, but motion information will remain similar within a scene [32]. The measure of the level and rate of change is used to detect change points within a sequence of frames. There are multiple types of motion information available for each frame. One type of motion information is the MB SoV, which measures the total motion within a MB [33]. Another type of motion information is the motion vector, which has been shown to be an effective indicator of change between a series of frames. By using the SoV and motion information, these measures can be used as an indicator of how similar a predicted frame is to its reference frame.

## Results and discussion

The results in this section demonstrate the application of automated change detection techniques to STEM videos. The sample videos are discussed, including the challenges presented in the videos and encoding parameters. Next the algorithm applied to the videos is explained. This covers any assumptions made about the data as well as any defined parameters. Finally, the results of the algorithm applied to the sample videos are shown.

### Sample videos

**Fig. 4:**
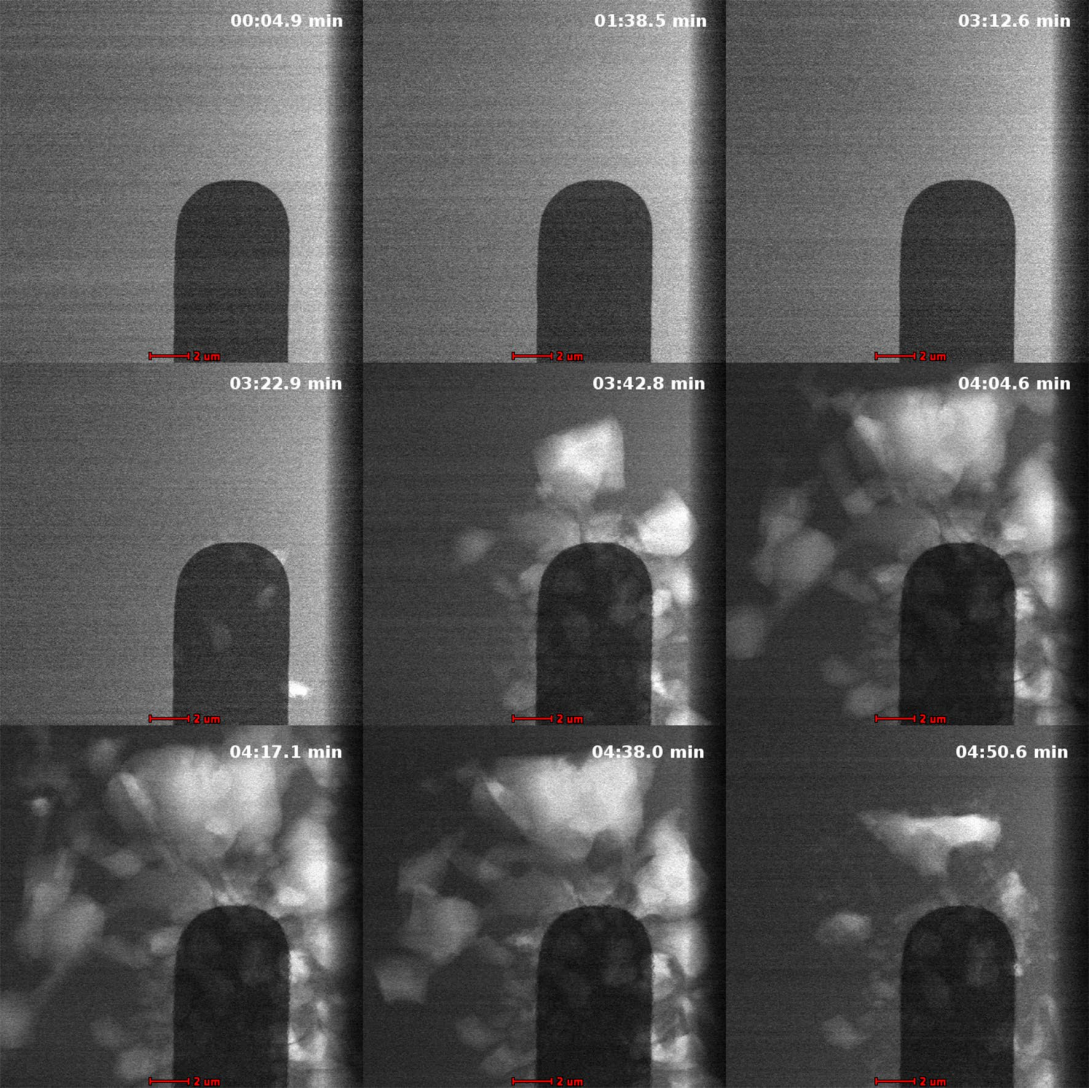
Summary of video transitions [8]

This technique is applied to two sample crystal growth videos. The two videos contain visually similar content; each starts with a series of nearly static frames, followed by rapid crystal growth, and finally gradual reduction. These growth and reduction occur over a series of frames. Frames from each of these transitions are shown in Fig. 4, which summarizes the three transitions taking place in the video. The first row of images shows the region of minimal change. The second row shows the growth over a series of frames. The third row shows a gradual reduction over time.

Before applying automated analysis, it is important to discuss the video-encoding parameters. These parameters must be carefully chosen so that the encoding algorithm produces output appropriate for analysis. The two sample videos in this case were encoded with the FFmpeg multimedia library. This library allows for full control over the video-encoding process through a series of parameters. The parameters chosen for this case encode the video as MPEG-2 using a constant frame rate (CFR). As opposed to CRF, variable frame rates (VFR) aim to eliminate similar content between frames in order to decrease the amount of data stored. Using CFR in this case reduces additional processing and allows for a fixed video quality level.

### Algorithm application

**Fig. 5:**
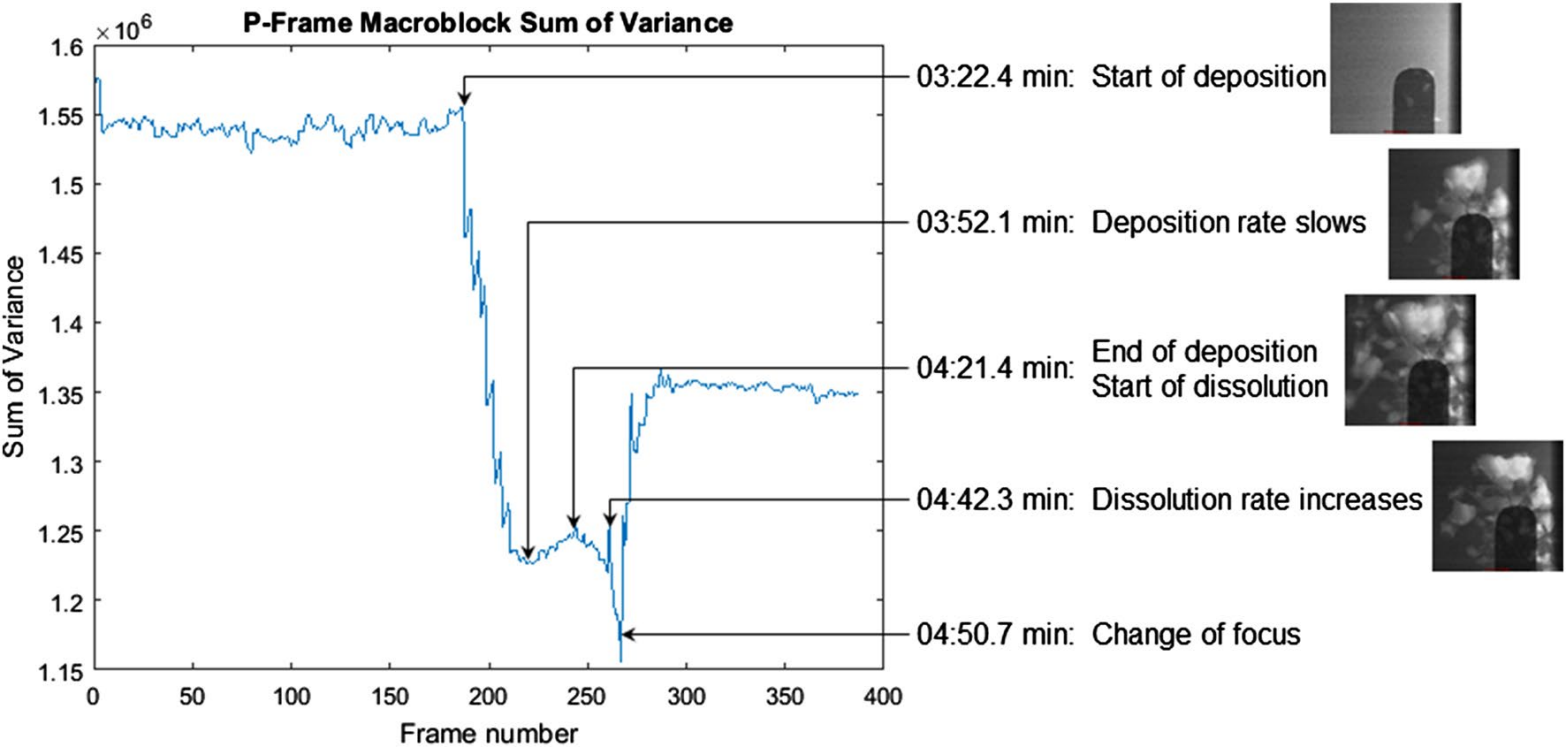
Macroblock sum of variance

Once the video has been encoded in the MPEG-2 format, the generated frame measurements previously discussed are now available for analysis. Of these, we will focus on the MB SoV and frame type. The total MB SoV for each P-frame is shown in Fig. 5.

Only the P-frames are considered in this case due to the inherent lack of motion information found in I-frames. Two visually distinguishable level changes occur in this sequence. Regions of static content remain roughly level, while rapid level changes indicate the presence of a change.

### Automated detection

**Fig. 6:**
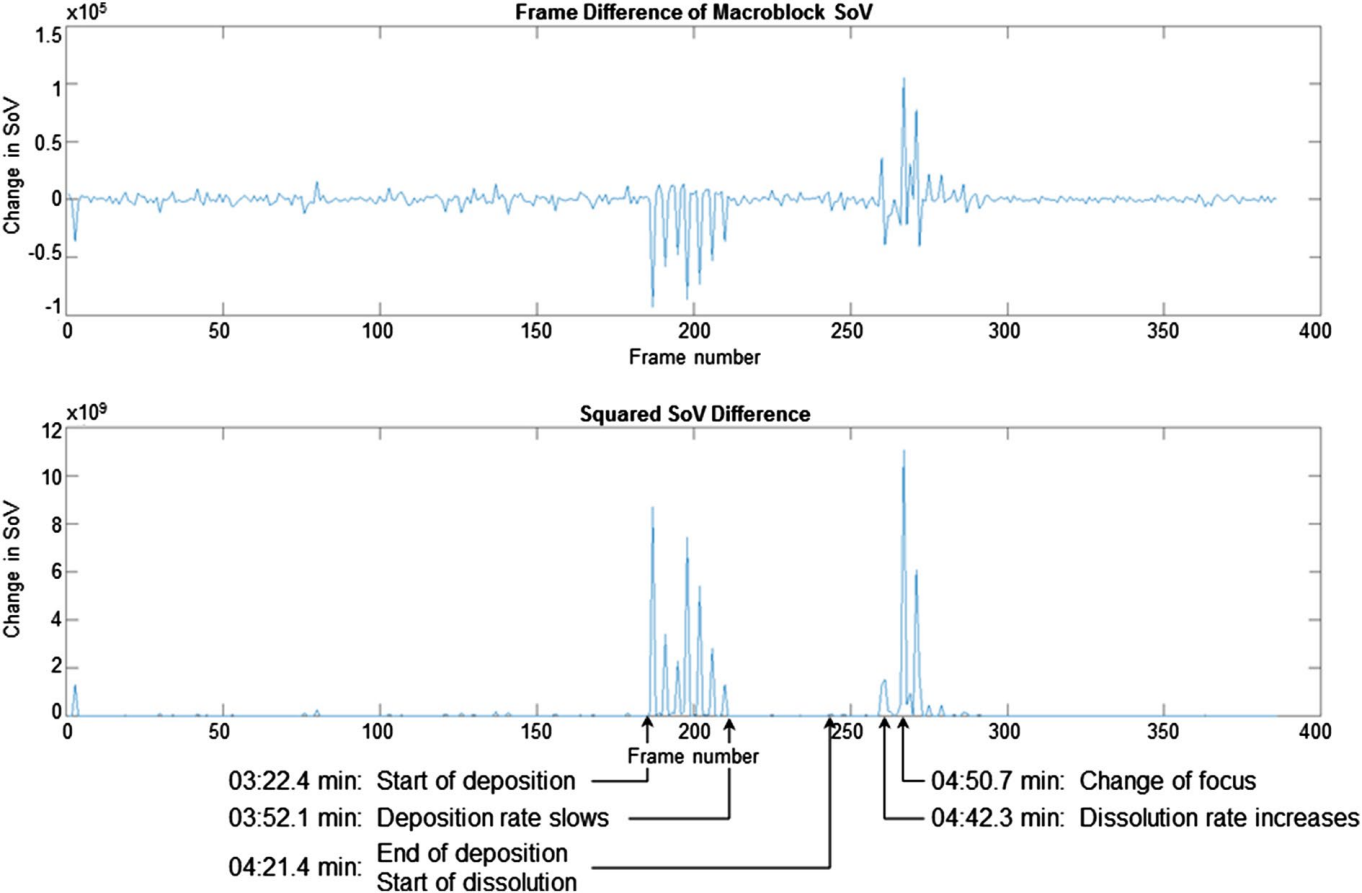
Difference in macroblock sum of variance compared to the square of the differences

Change is detected by examining sequential differences in MB SoV between P-frames. The difference signal is obtained by subtracting the SoV values of adjacent frames. This shows the amount of change occurring between consecutive frames, which is shown in Fig. 6. Regions showing large absolute difference correspond to the regions of change in the original signal.

In order to detect regions of change, it is necessary for background noise to be low so that transitions are easily distinguishable. To further reduce noise, we square the difference signal. Squaring the difference signal emphasizes the change while suppressing low-frequency noise. The result provides an absolute difference between frames. An example of the noise reduction compared to the original difference signal is shown in Fig. 6. The peaks in the difference signal make it possible to distinguish where transitions occur.

**Fig. 7:**
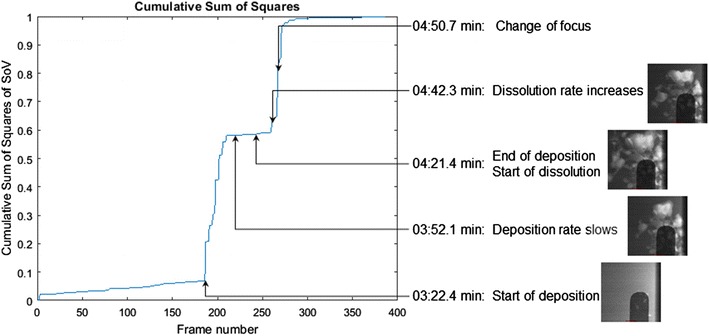
Cumulative sum of squared differences

Grouping frames into regions of similar content can be done by considering the total change. The cumulative sum of squared difference for each point provides a measure of the total change having occurred to a point. The sum of squared differences allows frames to be grouped based on the similarity of total change. This measure of the total change provides a simple method of identifying regions based on the similar levels. Figure 7 shows an example of the sum of squared differences for a video. Areas of little change remain flat, while changes will appear as rapid increases or jumps.

To find points best indicating where transitions occur, we need to define the characteristics of change. Between each frame, we are interested in how much change has taken place, which can be measured in two ways: the total distance between points and the angle of the vector relative to the independent axis. As each of these increases, the amount of measurable change also increases. To quantify this, we define a relevance measure for each pair of points [34].

$$\Delta y = y_{i} - y_{i + 1}$$

$$\theta = \tan^{ - 1} \;\Delta y$$

$$R = \left| {\theta \cdot \Delta y} \right|$$

For each pair of adjacent points, the relevance measure *R* is calculated. This measures the total change contributed by each of the components. The net change, denoted as ∆*y*, is the change in distance between points. Since the points measured by the sum of squared differences are the distance from the origin, the net change is the difference between the point values. Large distances between points indicate a large amount of change over this time. The angle is measured between the vector formed by the two points and the horizontal axis. In areas with little change, the sum of squared difference will be nearly flat which will result in angle near zero. For regions of large change, the signal increases rapidly resulting in angles near 90°.

### Algorithm results

Before automated analysis was performed, the frames in the video were manually reviewed for boundaries based on the visual change. These manually identified regions are listed in Table 1. There are three regions of change noted in the video.

**Table 1:** Annotated frames

Frame start	Frame end	Type
1	185	No change
186	242	Change (growth)
243	266	Change (shrinkage)
267	278	Change (background replacement)
279	387	No change

Automated analysis is performed based on the detection method previously described. Points of change are determined by applying a threshold to the values of *R* as defined above. The minimum threshold in this case is chosen as the 95th percentile. This detection algorithm is applied to the video with results recorded in Table 2. These results are consistent with the manually annotated results.

**Table 2:** Identified region boundaries

Frame start	Frame end
186	209
259	266
267	278

**Fig. 8:**
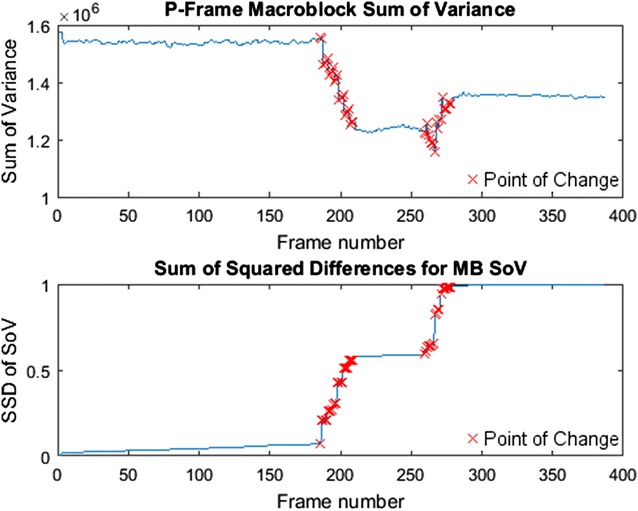
Sum of variance and sum of squared differences of SoV with change points identified

The algorithm identifies points of change that form a transition, while the regions between transitions can be grouped into areas of similar content. It can be seen that the algorithm identifies the critical regions where the most change takes place. These regions are identified in Fig. 8, which shows the points identified in the original signal as well as the squared sum of differences.

Points of change can be grouped together to form transition regions. These regions are formed by grouping together points of change occurring near one another. For this instance, changes found within ten frames of another change are used to form the region.

### Algorithm comparison

The technique described in this paper builds upon research in the area of shot boundary detection in the compressed domain. This analysis technique was chosen due to its execution speed and overall performance in detecting transitions. Other techniques exist which rely on methods such as machine learning, frame-based color histograms, and luminance values. While these techniques may have similar effectiveness in detecting changes, their runtime efficiency is significantly lower. Recent comparisons of techniques show that detection in the compressed domain can be done in less than real-time, while others require much more computational time [18].

## Conclusions

We have demonstrated that video analysis techniques used for shot boundary detection can be used to identify changes in the movies showing Li deposition/dissolution process in the in situ *ec*-STEM cell. Shot boundary detection offers a wide variety of techniques that can be applied to find points of change for different types of transitions and under different conditions. These methods allow for direct operation on compressed video without the need for full-frame decoding, which reduces the computational complexity. Metrics based on differences in motion between frames in MPEG video in the compressed domain are used. A metric is developed based on the total amount of change occurring at each point, which is used to identify transition regions. Experimental results show positive results for identifying the points where changes occur. These techniques could be applied to find transition points, which can aid in manual interpretation of the results, or potentially be applied to direct automatic frame capture.

### Future work

The video-encoding step produces a lossy signal which is typically avoided in the microscopy community. As such, this technique is strictly used as an automated means of detection. Future work may consider applying different compression algorithms, such as the latest H.264 standards. It may also be of interest to investigate other shot boundary detection algorithms that are more computationally expensive given that the analysis is a step performed independent of the experiment.

## Additional file

**Additional file 1.** Direct observation of Li dendrite formation in LiPF_6_ in PC/50 ppm H_2_O Pt working electrode in 1 M LiPF_6_ in PC electrolyte in the presence of 50 ppm H_2_O
